# Enhanced Macrophage Pannexin 1 Expression and Hemichannel Activation Exacerbates Lethal Experimental Sepsis

**DOI:** 10.1038/s41598-018-37232-z

**Published:** 2019-01-17

**Authors:** Weiqiang Chen, Shu Zhu, Yongjun Wang, Jianhua Li, Xiaoling Qiang, Xiaoling Zhao, Huan Yang, John D’Angelo, Lance Becker, Ping Wang, Kevin J. Tracey, Haichao Wang

**Affiliations:** 1 The Feinstein Institute for Medical Research, Northwell Health, 350 Community Drive, Manhasset, NY 11030 USA; 2 Zucker School of Medicine at Hofstra/Northwell, 500 Hofstra Blvd, Hempstead, NY 11549 USA; 3 Department of Pathology, New York University School of Medicine, 550 1st Ave, New York, NY 10016 USA

## Abstract

We have recently reported an important role of Connexin 43 (Cx43) hemichannels in the pathogenesis of lethal sepsis through facilitating ATP efflux to potentiate the double-stranded RNA-activated protein kinase R (PKR)-dependent macrophage activation. Here we further elucidated the possible role of Pannexin 1 (Panx1) hemichannel in lethal sepsis by assessing its expression along with the impact of a Panx1-specific mimetic inhibitory peptide, 10Panx, on macrophage hemichannel activity *in vitro* and animal sepsis lethality *in vivo*. Both crude bacterial lipopolysaccharide (LPS) and purified serum amyloid A (SAA) effectively induced the expression and extracellular release of Panx1 by macrophages or monocytes as judged by Western blotting and immunocytochemistry assays. In animal model of lethal sepsis, Panx1 expression levels were significantly elevated in the heart, but reduced in the kidney, lung, spleen, and blood. At relatively lower doses (10, 50, and 100 mg/kg), the Panx1 mimetic peptide, 10Panx, reproducibly exacerbated the sepsis-induced animal lethality, reducing survival rates from 60–70% to 0–10%. Consistently, 10Panx did not inhibit, but rather promoted, the LPS-induced elevation of Lucifer Yellow dye uptake, ATP release, and Nitric Oxide (NO) production. Collectively, these findings suggested that elevated macrophage Panx1 expression and hemichannel activation contribute to the pathogenesis of lethal sepsis.

## Introduction

Bacterial infections and sepsis are the most common causes of death in the intensive care unit, annually claiming >225,000 victims in the U.S. alone. The pathogenesis of sepsis remains poorly understood, but is partly attributable to dys-regulated inflammation propagated by macrophages and monocytes in response to microbial infections^1^. Macrophages and monocytes are equipped with various pattern recognition receptors (such as the Toll-like receptor 4, TLR4)^2^ to recognize distinct pathogen-associated molecular patterns [PAMPs, e.g., bacterial endotoxin (lipopolysaccharide, LPS)]^3^. Upon engagement of LPS by TLR4 receptor^2^, macrophages and monocytes sequentially release a wide array of “early” (e.g., TNF, IL-1β, IFN-γ, and CIRP)^4–7^, intermediate (e.g., serum amyloid A, SAA)^8^, and “late” proinflammatory mediators [e.g., nitric oxide (NO) and high mobility group box 1 (HMGB1)]^9–12^. Like exogenous bacterial endotoxin, some endogenous proinflammatory mediators (such as SAA) also use TLR4 to trigger the double-stranded RNA-dependent protein kinase R (PKR)-dependent inflammasome activation^13,14^, precipitating the subsequent release of late-acting proinflammatory mediators (e.g., NO and HMGB1)^8^.

Although ultrapure LPS can stimulate macrophages to produce early cytokines (e.g., TNF), it completely fails to induce NO production and HMGB1 secretion unless the LPS priming is accompanied by a second stimulus, ATP^15–19^. We recently reported that both crude LPS and purified SAA markedly stimulated macrophages to upregulate the expression of connexin 43 (Cx43) hemichannels, which provide one temporal mode of ATP release^20^. However, the expression profile of another class of hemichannel protein, Pannexin 1 (Panx1), during lethal sepsis remains poorly understood.

Pannexins were first discovered in flatworm by Panchin *et al*. as a class of innexin-like membrane channels that form gap junctions in the invertebrate animals. They were named as pannexins (‘pan’, meaning ‘all’ in Greek) to reflect their widespread expression in many vertebrate species^21^. Unlike their invertebrate counterparts, the vertebrate pannexins assemble into hexameric hemichannels that allow ATP release from many types of cells^22,23^. The Panx family consists of 3 members: Panx1 is ubiquitously expressed in most tissues [e.g., the eye, thyroid, prostate, kidney, liver, immune cells, and central nervous system (CNS)]; Panx2 is primarily restricted to the CNS; and Panx3 is predominantly found in the skin and skeletal tissues^24^. Under physiological conditions, Panx1 hemichannels remain largely closed, as its C-terminal tail may physically plug up the hemichannel pore from the cytoplasmic side. In response to inflammatory stimuli, its C-terminal tail could be cleaved by several caspases, resulting in a progressive opening of Panx1 hemichannel and extracellular release of larger molecules like ATP^25^, which activates P2X7R to trigger inflammasome activation^26^, HMGB1 release and NO production^17,27^. In microglia, the expression of Panx1 hemichannels is inducible by TNF, IL-1, and IFN-γ^24^, but its expression in other innate immune cells (e.g., macrophages) remains poorly elucidated.

In keeping with its broad distribution, Panx1 hemichannel has now been implicated in a wide variety of pathological conditions by the use of mimetic inhibitory peptides. The 10Panx is a synthetic peptide that mimics a sequence (WRQAAFVDSY) in the second extracellular loop of Panx1, and can inhibit the Panx1 hemichannel-mediated ATP release and inflammasome activation in astrocytes and macrophages^28–30^. *In vivo*, pharmacological blockade of Panx1 hemichannels with 10Panx peptide attenuated the progression of acetaminophen-induced hepatotoxicity^31^, neuropathic pain^32^ and epilepsy^33^. Notably, the hemichannel inhibitory properties of 10Panx was mostly demonstrated at relatively higher concentrations (300–500 µM; or 370–620 µg/ml)^33–35^, and less obvious when given at lower concentrations (e.g., 100 µM; or 125 µg/ml)^36^. In addition, the genetic disruption of Panx1 expression rendered animals less susceptible to lethal endotoxemia^26^ or cerebral ischemic injury^37^, but surprisingly more susceptible to lethal sepsis induced by a surgical procedure termed cecal ligation and puncture (CLP)^38^. It is thus important to assess how 10Panx affects the outcomes of lethal sepsis and macrophage hemichannel activation if given at relatively lower concentrations. Therefore, here we sought to examine whether Panx1 protein was inducible in macrophage cultures, and how 10Panx influences macrophage hemichannel activation *in vitro* and affects the sequelae of lethal sepsis *in vivo*.

## Results

### Exogenous endotoxin and endogenous SAA induced Panx1 expression and release in macrophage and monocyte cultures

**Figure 1:**
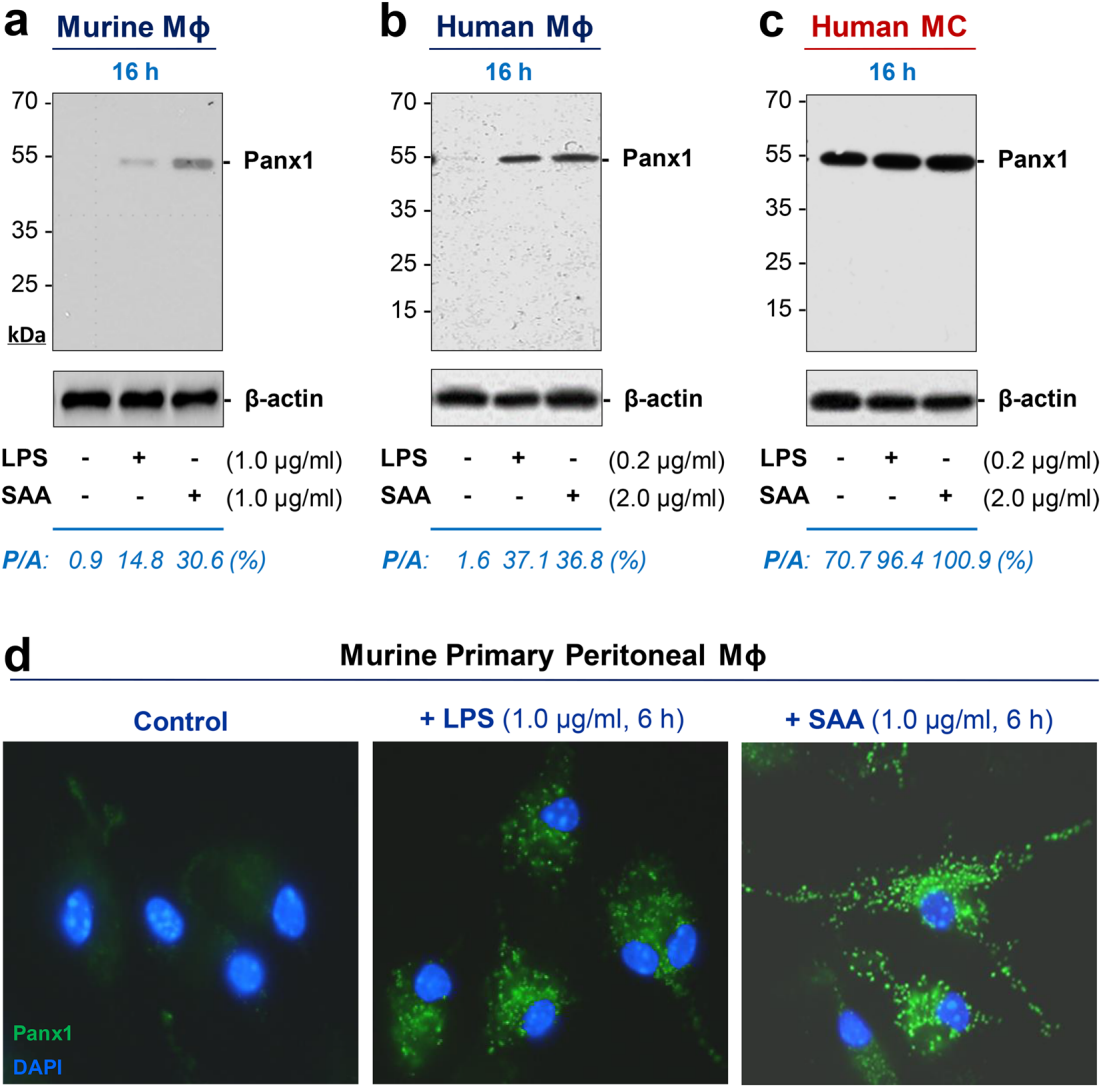
An exogenous bacterial endotoxin (LPS) and an endogenous inflammatory cytokine (SAA) up-regulated Panx1 expression in macrophage (Mϕ) and monocyte (MC) cultures. Murine macrophage-like RAW264.7 cells (Panel a), differentiated human macrophages (Panel b), human peripheral blood mononuclear cells (MC, Panel c), or primary murine peritoneal macrophages (Panel d) were stimulated with crude LPS or SAA at indicated concentrations for indicated time periods, and the cellular Panx1 levels were measured by Western blotting (Panel a,b,c) or immunocytochemistry (Panel d), respectively. A house-keeping gene product β-actin was used as a loading control to estimate the relative Panx1/β-actin (P/A) ratio in macrophage or monocyte cultures.

To assess the potential regulatory role of Panx1 hemichannels in innate immunity, we first characterized Panx1 expression in murine and human macrophage cultures following stimulation with a bacterial endotoxin (LPS) or an inflammatory cytokine (SAA). Although quiescent murine and human macrophages constitutively expressed Panx1 at relatively low levels (Fig. 1a,b), crude LPS and purified SAA induced a dramatic elevation of Panx1 protein levels in these innate immune cells. This upregulation of Panx1 was confirmed in primary murine peritoneal macrophages by immunocytochemistry (Fig. 1d), which revealed a marked increase in the Panx1-specific punctate immunostaining post LPS or SAA stimulation. Taken together, these results suggest that both exogenous and endogenous inflammatory stimuli induce similar elevation of cellular Panx1 protein levels in macrophage cultures.

**Figure 2:**
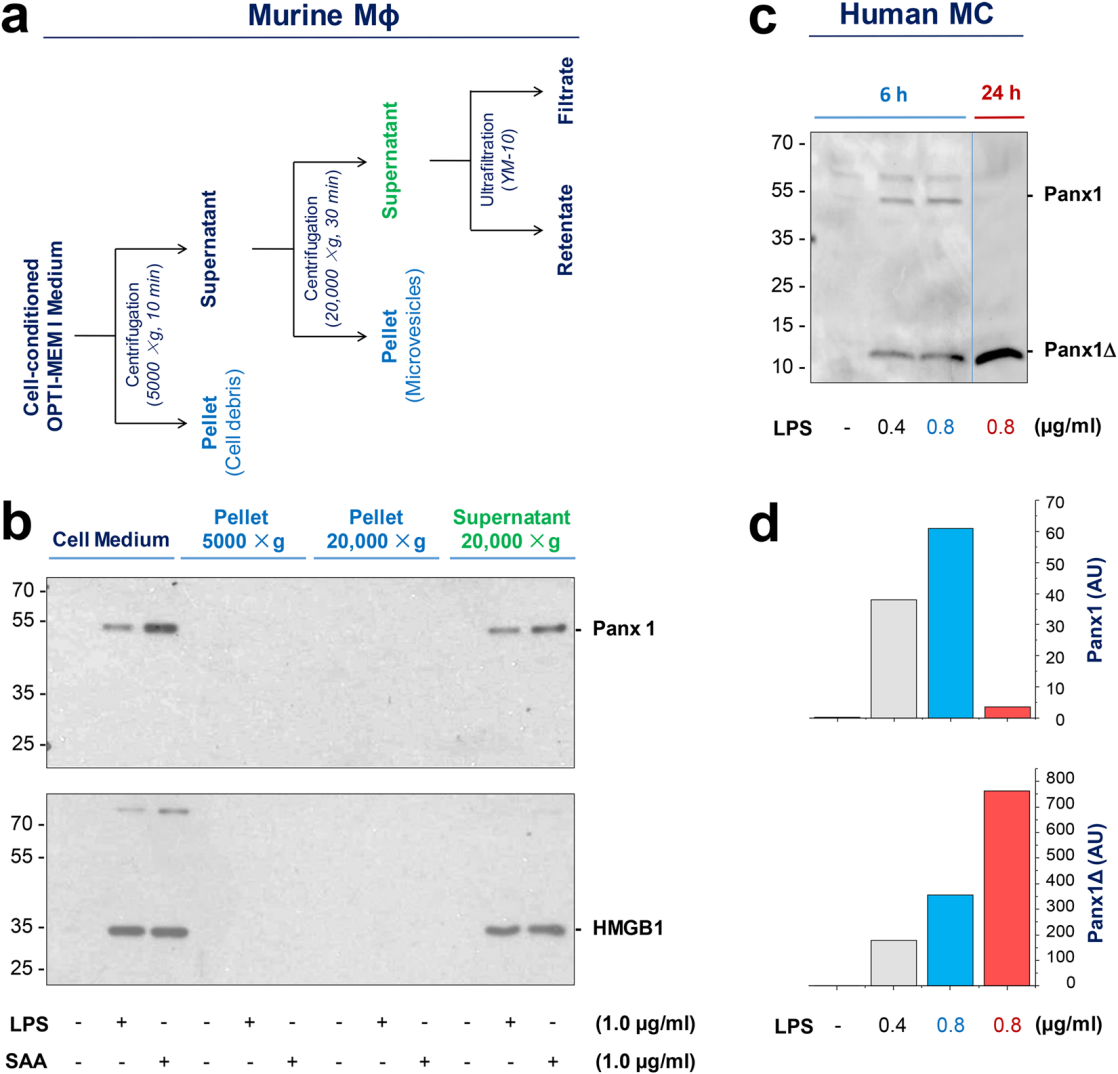
Crude LPS and purified SAA induced Panx1 release in macrophage and monocyte cultures. (**a**,**b**) Extracellular release of Panx1 by murine macrophages. Murine macrophage-like RAW264.7 cells were stimulated with LPS or SAA at indicated concentrations for 16 h. The macrophage-conditioned culture medium was subjected to differential centrifugations, and each fraction was assayed for Panx1 by Western blotting analysis. Note Panx1 was found in the LPS- or SAA-stimulated macrophage-conditioned medium (“Cell Medium”), and the supernatant of the 20,000 × g centrifugation. (**c**,**d**) Extracellular release of Panx1 by primary human monocytes. Human PBMCs were stimulated with crude LPS at indicated concentrations for different time periods, and extracellular Panx1 levels were determined by Western blotting analysis. The relative optical intensity of the 48-kDa (Panx1) and 12-kDa (Panx1Δ) band was measured, and expressed as an arbitrary unit (AU).

To understand the mechanism underlying the regulation of Panx1 expression in activated macrophages, we also tested whether Panx1 was released into the extracellular milieu. Surprisingly, both crude LPS and SAA induced a marked and parallel release of Panx1 and HMGB1 within 16 h of stimulation (Fig. 2b). To determine whether extracellular Panx1 or HMGB1 was associated with membrane-containing cell debris or microvesicles, the macrophage-conditioned culture medium was subjected to differential centrifugations (Fig. 2a), and each fraction was immunoblotted with Panx1- or HMGB1-specific antibodies. Like extracellular HMGB1, the released Panx1 was not found in the cell debris or microvesicle fractions, but was predominantly retained in the supernatant of ultracentrifugation (20,000 × g) (Fig. 2b). These data suggest that LPS and SAA induced a parallel Panx1 upregulation and secretion in innate immune cells.

In a sharp contrast to macrophage cultures, quiescent human monocytes expressed Panx1 at relatively abundant levels (Fig. 1c). In response to LPS or SAA stimulation, there was a similar but marginal (35–40%) increase of cellular Panx1 content in monocyte cultures (Fig. 1c). Furthermore, following endotoxin stimulation, human monocytes also markedly released Panx1 in a time-dependent fashion (Fig. 2c). The LPS-induced Panx1 release was obvious within 6–16 h (Fig. 2c), but gradually diminished 24 h after stimulation (Fig. 2c). We did not sequence the 48-kDa band, because it closely matched with the predicted molecular weight of Panx1, and was specifically recognized by two independent monoclonal and polyclonal antibodies in both macrophage (Figs 1a,b; and 2b) and monocyte (Figs 1c and 2c) cultures.

Notably, the extracellular release of Panx1 was accompanied by the appearance of a smaller molecular weight (12 kDa) band (Fig. 2c) that was recognized by a specific monoclonal antibody against the C-terminus of human Panx1. We did not sequence this 12-kDa band, because it was specifically detected by the same monoclonal antibody only in the monocyte-conditioned culture medium (Fig. 2c), but not in monocyte whole-cell lysate (Fig. 1c). Furthermore, the anti-parallel changes of the 48-kDa (Panx1) and 12-kDa (Panx1Δ) band intensities (Fig. 2c,d) supported a possibility that the lower molecular weight band was reminiscent of the C-terminal fragment of Panx1 degradation products. To elucidate the possible role of extracellular Panx1, it might be necessary to confirm the identity of the 12-kDa band by sequencing analysis particularly following partial enrichment by immunoprecipitation.

### Altered Panx1 expression in lethal sepsis

**Figure 3:**
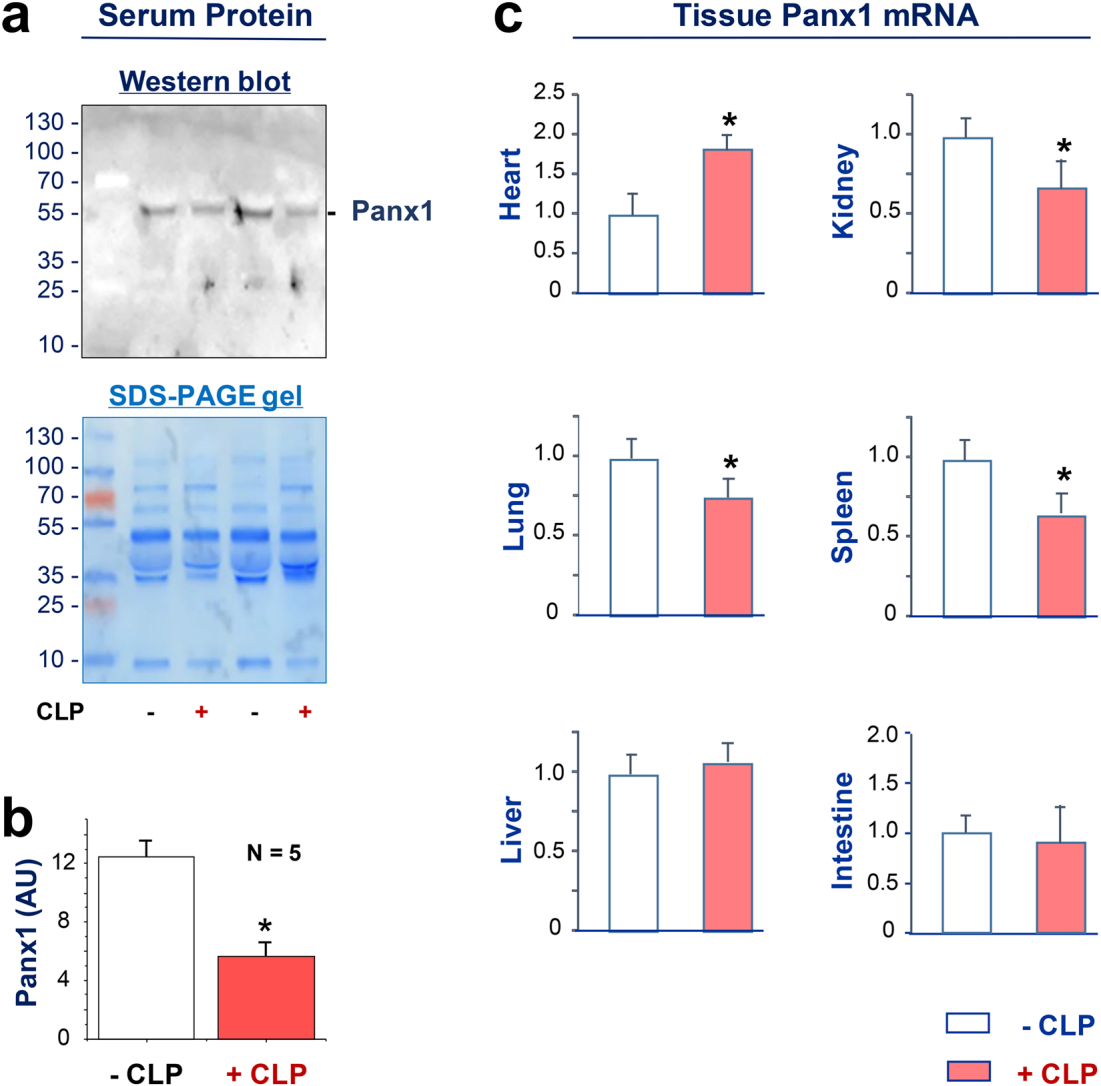
Altered expression of Panx1 during lethal sepsis. Balb/c mice were subjected to lethal sepsis by a surgical procedure termed cecal ligation and puncture (CLP), blood and various tissues were harvested at 24 h post CLP to measure Panx1 protein (Panel a,b) or mRNA levels (Panel c) by Western blotting and real-time RT-PCR, respectively. Serum Panx1 levels were measured by the optical intensity of the 48-kDa band on the Western blot, and expressed as the % of the optical band intensity of corresponding total proteins on SDS-PAGE gels. *P < 0.05 versus normal “−CLP” control.

To understand the role of Panx1 in lethal sepsis, we examined the possible change in its expression levels using a clinically relevant animal model of lethal sepsis induced by a surgical procedure termed cecal ligation and puncture (CLP). Although Panx1 was readily detectable in the serum of normal healthy female mice (Fig. 3a), its circulating levels were significantly reduced at 24 h post the onset of sepsis (Fig. 3a,b). To assess Panx1 expression profile in other tissues, we measured its mRNA levels by real-time RT-PCR technique. Although CLP did not significantly alter Panx1 mRNA expression in the intestine and liver at 24 h post CLP, it significantly reduced Panx1 expression in the kidney, lung and spleen (Fig. 3c). In a sharp contrast, CLP significantly increased Panx1 mRNA expression levels in the heart (Fig. 3c), suggesting a tissue-specific divergent regulation of Panx1 during lethal sepsis.

### Panx1 mimetic peptide 10Panx worsened the outcome of lethal sepsis

**Figure 4:**
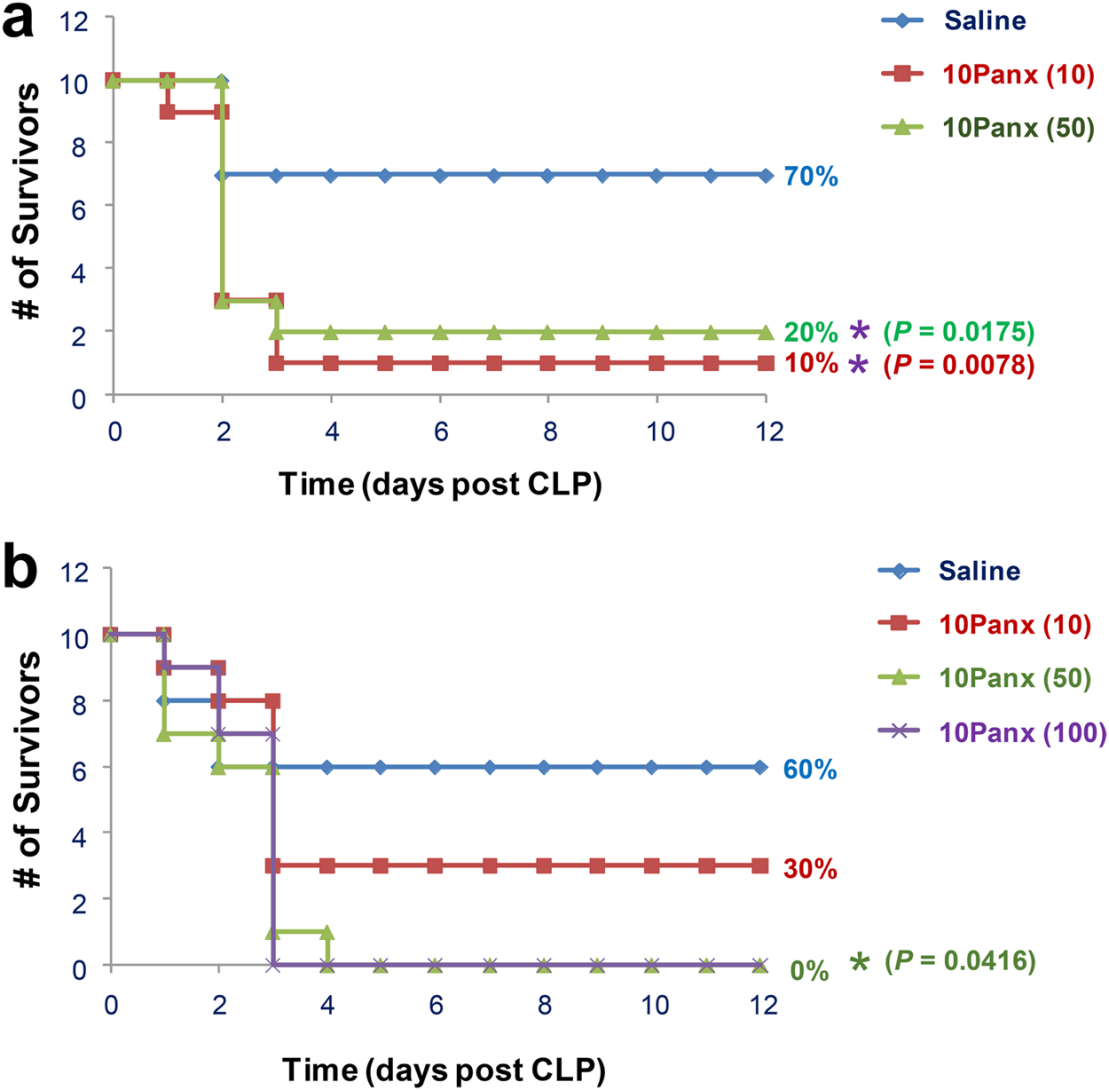
Panx1 mimetic peptide (10Panx) exacerbated lethal sepsis. Male Balb/c mice were subjected to CLP-induced sepsis, and intraperitoneally administered with control saline (0.2 ml/mouse) or 10Panx peptide at indicated doses at +2, +24 h post CLP. Animal survival was assessed for up to two weeks, and the Kaplan-Meier method was used to compare the differences in mortality rates between groups. *P < 0.05 versus “+Saline” group.

To assess the role of Panx1 in lethal sepsis, we examined the impact of Panx1 mimetic peptide on the outcome of CLP-induced lethal sepsis. Unlike the scrambled control peptide (FSVYWAQADR), which did not alter the animal survival rate as compared with saline controls (65% for the scrambled peptide versus 65% for the saline control, N = 20 mice), the 10Panx (WRQAAFVDSY) reproducibly decreased animal survival rate from 65% to 45% (N = 20 mice, *summary of two independent experiments with similar results*) when both peptides were given at a relatively higher dose (120 mg/kg). To verify this deleterious effect, we obtained 10Panx peptide from another commercial source (GenScript), and further tested it using multiple lower doses. As shown in Fig. 4, at relatively lower doses (10, 50 mg/kg), the 10Panx reproducibly worsened the outcome of lethal sepsis, significantly decreasing animal survival rates from 60–70% in the control group to 0–10% in the treatment group (Fig. 4a,b).

### 10Panx enhanced the LPS- and SAA-induced hemichannel activation

**Figure 5:**
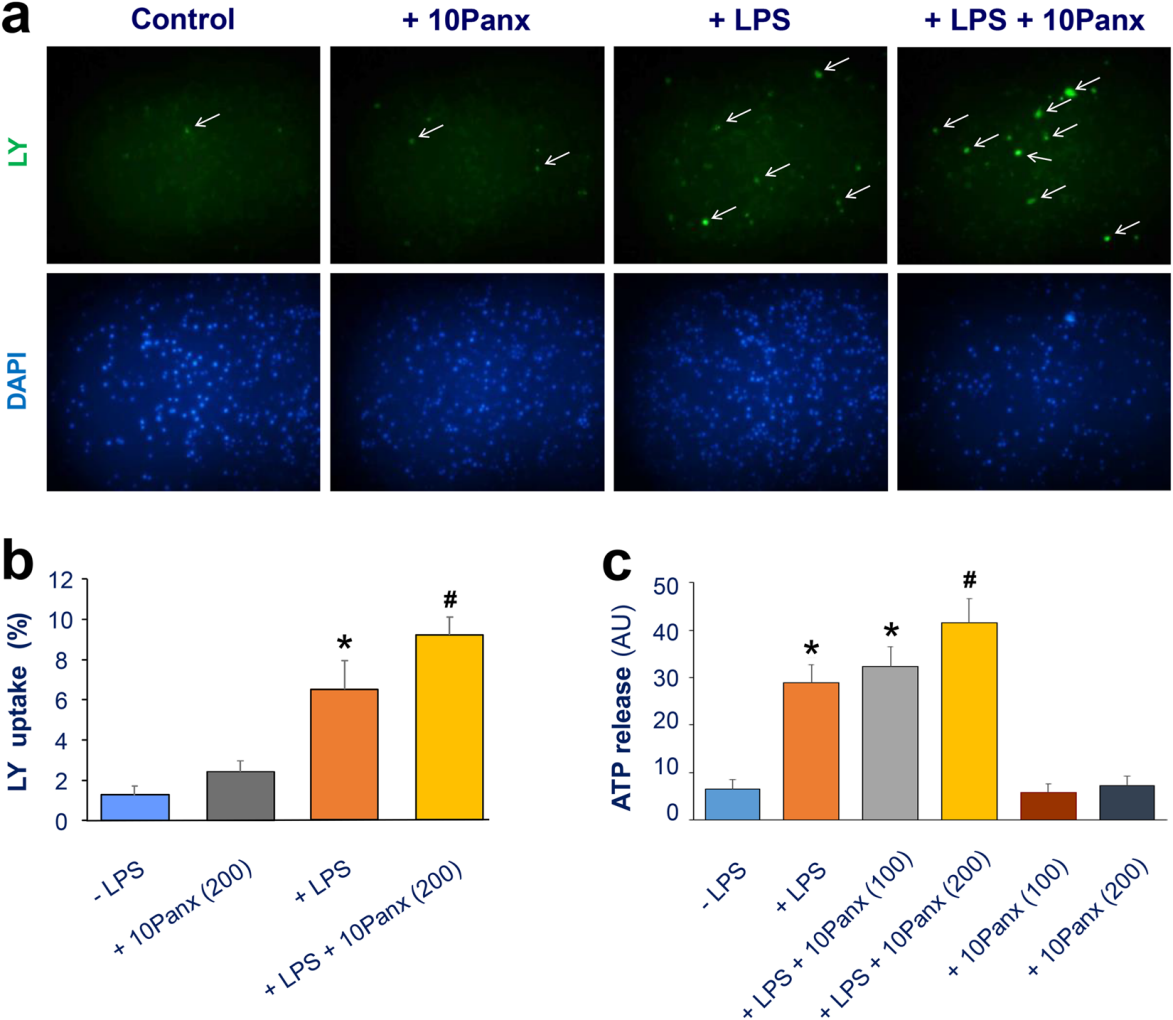
Panx1 mimetic peptide 10Panx enhanced LPS-induced macrophage hemichannel activation. (**a**,**b**) 10Panx elevated the LPS-induced Lucifer Yellow dye uptake. RAW264.7 cells were stimulated with LPS (1.0 μg/ml) in the absence or presence of 10Panx at indicated concentrations for 16 h, and subsequently incubated with Lucifer Yellow (LY, 1.0 mg/ml) for 15 min. Following fixation and three extensive washes, the number of cells with diffused fluorescent signals was counted under a fluorescence microscope, and expressed as a percentage of total cell numbers (DAPI-stained nuclei) in six fields. (**c**) 10Panx enhanced the LPS-induced ATP release. RAW264.7 cells were cultured in serum-free DMEM medium, and stimulated with LPS (1.0 μg/ml) in the absence or presence of Panx1 mimetic peptide at indicated concentrations, and the cell-conditioned culture medium was collected and subjected to ATP measurement. *P < 0.05 versus “-LPS” Control; ^#^*P* < 0.05 versus “+LPS” control.

Although it was previously shown that 10Panx could inhibit hemichannels at relatively higher concentrations (300–500 µM; or 370–620 µg/ml)^33–35^, it is not yet known how 10Panx affects macrophage hemichannel activities when given at lower concentrations. As expected, crude LPS caused a marked increase in the percentage of LY-positive cells (Fig. 5a,b), suggesting an elevation of Panx1 hemichannel activities. When given at relatively lower concentrations (200 µg/ml; or 161 µM), 10Panx itself did not significantly affect the LY dye uptake (Fig. 5a,b). Conversely, the pre-incubation of macrophage cultures with the 10Panx peptide at this concentration (200 µg/ml) resulted in a significant enhancement of the LPS-induced elevation of LY dye uptake (Fig. 5a,b).

Because Panx1 hemichannels also provide a mode of ATP efflux from activated innate immune cells, we examined how 10Panx peptide affected the endotoxin-induced ATP release. Although 10Panx itself did not affect ATP release, it dose-dependently elevated the LPS-induced ATP release (Fig. 5c). It suggests that at relatively lower concentrations, 10Panx significantly enhanced the LPS-induced hemichannel activation as judged by the parallel elevation of LY dye uptake and ATP release.

### Divergent impacts of 10Panx on the LPS- and SAA-induced NO production and cytokine release

**Figure 6:**
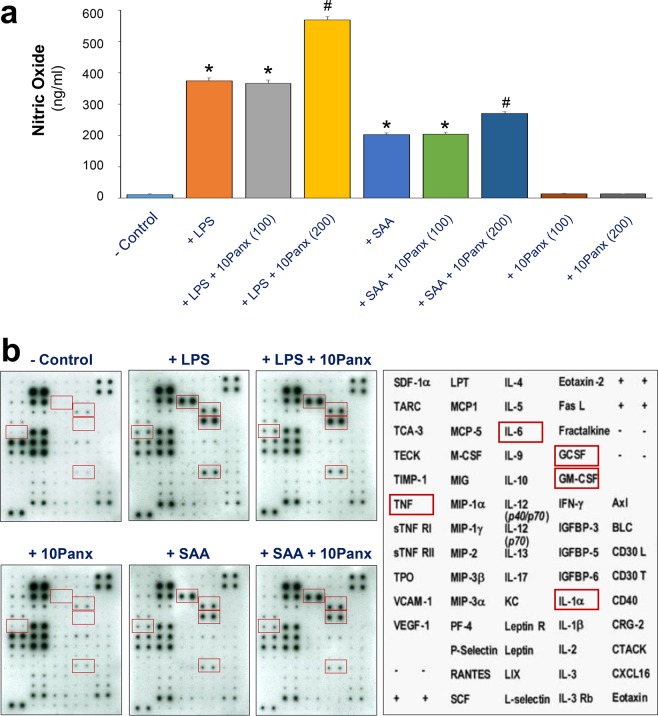
10Panx specifically enhanced the LPS- and SAA-induced NO production. RAW264.7 cells were stimulated with LPS (1.0 μg/ml) or SAA (1.0 μg/ml) for 16 h in the absence or presence of 10Panx at indicated concentrations, and the levels of NO (Panel a) and various cytokines (Panel b) were determined by the Griess reaction or Cytokine Antibody Array, respectively. *P < 0.05 versus “−LPS” Control; ^#^*P* < 0.05 versus “+LPS” or corresponding “+SAA” controls.

Although both crude LPS and purified SAA can stimulate macrophages to release a wide array of proinflammatory mediators, the maximal induction of NO is dependent on the availability of extracellular ATP^16,17,27^, as well as the resultant activation of PKR^8,39^. We thus tested the effects of 10Panx on the crude LPS- and purified SAA-induced production of NO and other cytokines. Although 10Panx significantly enhanced the LPS- and SAA-induced NO production (Fig. 6a), it did not markedly affect the LPS- or SAA-induced release of most other cytokines (e.g., G-CSF, GM-CSF, and IL-6) in macrophage cultures (Fig. 6b). It supports an important role for Panx1 in LPS- and SAA-induced ATP efflux and resultant iNOS up-regulation and NO production.

## Discussion

Monocytes originate from bone marrow hematopoietic stem cells, and continuously circulate in the blood to search for invading pathogens or damaged tissues. In response to infection or injury, they can infiltrate into infected/injured tissues, and differentiate into tissue-specific resident macrophages. The pathogenesis of sepsis is partly attributable to dys-regulated inflammation propagated by both macrophages and monocytes in response to pathogen-associated molecular patterns (PAMPs, e.g., bacterial endotoxin) or host-derived inflammatory mediators (such as SAA and NO)^8^. We previously reported that crude LPS and highly purified SAA could upregulate the expression of Cx43 hemichannels, which facilitate ATP release to trigger PKR-dependent macrophage activation^40^. In the present study, we demonstrated that LPS and SAA similarly upregulated the expression and extracellular release of Panx1 in macrophage and monocyte cultures. Although circulating monocytes expressed Panx1 abundantly, once reached extravascular tissues and terminally differentiated, these tissue-specific resident macrophages appeared to markedly down-regulate its expression levels. In response to inflammatory stimuli, macrophages then effectively upregulate Panx1 expression, thereby facilitating the release of inflammatory signaling molecules (such as ATP) to orchestrate a pronounced inflammatory response. Our findings were in agreement with a previous report that the expression of Panx1 hemichannels was inducible in brain-resident macrophages (microglia) by a variety of proinflammatory cytokines such as TNF, IL-1, and IFN-γ^24^.

Differential centrifugation analysis of macrophage-conditioned culture medium revealed that the extracellular Panx1 was not associated with cell debris or microvesicles. It is currently not known whether extracellular Panx1 exists in other smaller membrane-associated structures, or can be trafficked back to cytoplasmic membranes of innate immune cells. It will thus be important to verify possible Panx1 extracellular trafficking – detachment from or re-localization back to, macrophage cytoplasmic membranes, using biotinylation techniques^41,42^. For instance, activated immune cells or Panx1 protein (isolated from the activated macrophage-conditioned culture medium) can be biotinylated using EZ-link NHS-LC-Biotin or EZ-link Sulfo NHS-LC biotin, and the possible trafficking of biotinylated Panx1 will be monitored using streptavidin-conjugated fluorescent probes. If biotinylated Panx1 could be detached from and/or re-localized back to the cytoplasmic membrane of innate immune cells, it will suggest a possible role of extracellular Panx1 in inflammatory diseases.

The expression of Cx43 and Panx1 hemichannel proteins might be differentially regulated in distinct tissues. For instance, endotoxin reduced Cx43 expression in the liver and heart^43^, but increased its expression in the kidney, lung^44^, and macrophages^40,45,46^. Here we demonstrated that during experimental sepsis, Panx1 mRNA expression was significantly elevated in the heart, but decreased in the kidney, lung, and spleen. These findings have confirmed the broad distribution of Panx1 in various types of tissues, and suggested that different hemichannel proteins (e.g., Cx43 and Panx1) may be divergently regulated during endotoxemia and microbial infections. It supports a possibility to develop both Cx43- and Panx1-sepcific inhibitors to strategically attenuate compartmentalized dysregulated inflammatory responses in lethal systemic inflammation. The parallel reduction of Panx1 protein levels in the circulation and its mRNA levels in several macrophage-rich organs suggested that the lung and spleen may partly contribute to systemic Panx1 accumulation under physiological conditions. It will thus be important to definitively determine the cellular source of circulating Panx1 in separate studies.

To modulate the hemichannel activities, various mimetic peptides have been designed to mimic extracellular or intracellular loops of Cx43 and Panx1. We recently found that two different Cx43 mimetic peptides, the GAP26 and TAT-GAP19, distinctly affected the LPS-induced hemichannel activation *in vitro*, and divergently altered the sequelae of lethal sepsis *in vivo*^40^. Here we demonstrated that a Panx1-specific mimetic inhibitory peptide, 10Panx, reproducibly exacerbated the CLP-induced animal lethality particularly when given at relatively lower doses (10–50 mg/kg; equivalent to 8–40 µM). Because our findings seemingly contradicted with previously reported protective effects of 10Panx in other animal models of diseases (e.g., brain ischemic injury, liver toxemia injury, neuropathic pain, and epilepsy)^31,32,47,33^, we exhaustively confirmed this finding using 10Panx peptide from different sources. At a wide range of doses, 10Panx from two independent sources reproducibly worsened the outcome of lethal sepsis, confirming a deleterious effect of 10Panx in experimental sepsis.

Because the hemichannel inhibitory properties of 10Panx was previously demonstrated at relatively higher concentrations (300–500 µM; or 370–620 µg/ml)^33–35^, here we tested the possibility that 10Panx may adversely promote macrophage hemichannel activation if given at relatively lower concentrations. We employed both LY dye uptake and ATP release techniques to measure macrophage hemichannel activities. Indeed, at relatively lower concentrations, 10Panx significantly enhanced the LPS-induced LY uptake and ATP release, although 10Panx itself failed to affect macrophage hemichannel activities in the absence of other inflammatory stimuli. Under similar experimental conditions, we have confirmed reproducible inhibitory effects of other well-known hemichannel blockers [e.g., carbenoxolone (CBX), brilliant blue G (BBG), oxidized ATP (oATP), and GAP26] on the LPS-induced macrophage hemichannel activation^8,40^, which provided positive controls for the present experimental setting. Our current finding was also in agreement with a recent report that 10Panx could not inhibit macrophage hemichannel activities when given at a relatively lower concentration (e.g., 100 µM)^36^. It suggested that the 10Panx’s macrophage hemichannel-enhancing properties *in vitro* correlated with its deleterious impact on lethal sepsis *in vivo*, enforcing the notion that excessive macrophage Panx1 hemichannel activation contributes to the pathogenesis of lethal systemic inflammation^26^.

Although Panx1 channels remain largely closed under physiological conditions, the cleavage of its C-terminal tail by various caspases following inflammatory stimulation enables a progressive opening of Panx1 channel and flux of larger molecules like ATP^25^. Consistent with the notion that the enzymatic cleavage of Panx1 is required for the opening of Panx1 hemichannel^25^, we found that the extracellular release of Panx1 was accompanied by the appearance of a smaller molecular weight product, possibly reminiscent of a Panx1 degradation product. In agreement with the essential role of ATP in PKR-dependent inflammasome activation^8,18,26^ and the resultant NO synthesis^16,17,27,39^, we found that 10Panx significantly elevated the LPS- or SAA-induced NO production. Our findings were consistent with previous observations that extracellular ATP potentiated the LPS-induced iNOS expression and NO production^16,17,27^, and support the notion that hemichannel-dependent ATP release might enhance the PKR-dependent iNOS production in macrophage cultures.

In summary, here we demonstrated that both LPS and SAA induced a time-dependent production and extracellular release of Panx1 in innate immune cells. In animal model of experimental sepsis, Panx1 expression was significantly elevated in the heart, and a Panx1-specific mimetic peptide 10Panx reproducibly exacerbated the outcome of lethal sepsis. The harmful effects of 10Panx correlated to its enhancement of the LPS- or SAA-induced hemichannel activation, ATP release and NO production, enforcing the notion that excessive macrophage Panx1 hemichannel activation may contribute to the pathogenesis of lethal systemic inflammation. Although we now propose a regulatory role of macrophage-associated Panx1 in the regulation of ATP release and immune activation, our present findings will likely stimulate extensive interest in further elucidating the possible role of extracellular Panx1 in inflammatory diseases. In addition, our study suggests that caution should be exercised when using various mimetic peptides to interfere with macrophage hemichannel activities at different dose regimens and experimental conditions.

## Material and Methods

### Materials

Crude bacterial endotoxin (lipopolysaccharide, LPS, *E. coli 0111:B4*), paraformaldehyde (PF, P6148), Lucifer Yellow (L0144), human serum (H3667), mouse anti-β-actin antibodies (A1978) were obtained from Sigma-Aldrich (St. Louis, MO, USA). A Panx1 mimetic peptide, 10Panx (WRQAAFVDSY, MW = 1242.37; Cat. No. 3348) and a Scrambled control peptide (FSVYWAQADR, MW = 1242.37; Cat. No. 3708) were purchased from the Tocris (Bio-Techne Corporation, MN, USA). Alternatively, 10Panx was also synthesized at >95% purity by GenScript (Piscataway, NJ, USA). As previously described^40^, recombinant human SAA (also termed Apo-SAA, *Cat. No*. 300–13) was purchased from PeproTech (Rocky Hill, NJ). The SAA is almost identical to human SAA1α, except for the presence of an N-terminal Met, the substitution of Asn for Asp at position 60, and Arg for His at position 71. Dulbecco’s modified Eagle medium (DMEM, 11995-065) and penicillin/ streptomycin (cat. 15140-122) were from Invitrogen/Life Technologies (Carlsbad, CA, USA). Human macrophage colony-stimulating factor (M-CSF, Cat. SRP-3110) was purchased from Peprotech (Rocky Hill, NJ, USA). Fetal bovine serum was obtained from Crystalgen (FBS-500, Commack, NY, USA) and heat-inactivated before use. OPTI-MEM® I Reduced-Serum Medium (Cat. #31985062) was obtained from the ThermoFisher Scientific (Springfield Township, NJ, USA). Anti-HMGB1 antibody was antigen-affinity-purified from serum of rat HMGB1-immunized rabbits as previously described^11^. Rabbit monoclonal antibody (Cat. # ab124969) against the C-terminal region (residue 350 to the C-terminus) of human Panx1 or rabbit polyclonal antibody (Cat. # ab139715) against N-terminal region of human Panx1 were obtained from Abcam (Cambridge, MA, USA). HRP conjugated donkey anti-rabbit IgG was from GE Healthcare (NA934; Port Washington, NY, USA). Balb/c male or female mice with age of 7–8 weeks were obtained from Taconic Biosciences (Hudson, NY, USA). Macrophage cell line RAW264.7 was obtained from the American Type Culture Collection (ATCC, Rockville, MD, USA).

### Cell culture

Primary peritoneal macrophages were isolated from young male Balb/c (7–8 wks, 20–25 g) at 3 days after intraperitoneal injection of 2 ml thioglycollate broth (4%) as previously described^48,49^. Briefly, mice were sacrificed by CO_2_ asphyxiation, and the abdomen region was immediately cleaned with 70% ethanol before making a small excision to expose the abdominal wall, and to insert a catheter into viscera-free pocket to wash out peritoneal macrophages with sucrose solution (11.6%, 7.0 ml). Human blood was purchased from the New York Blood Center (Long Island City, NY, USA), and human peripheral blood mononuclear cells (HuPBMCs) were isolated by density gradient centrifugation through Ficoll (Ficoll-Paque PLUS, Pharmacia, Piscataway, NJ, USA) as previously described^50–52^. To differentiate human monocytes into macrophages, human PBMCs were cultured in RPMI 1640 medium with 10% heat- inactivated human serum for two hours to allow cell adhesion. Afterwards, adherent cells were detached with 10 mM EDTA, re-suspended (10^6^ cells/ml) in medium supplemented with human M-CSF (20 ng/mL), and cultured for 6 days. As previously described^40^, macrophages and monocytes (HuBPMCs) were cultured in DMEM supplemented with 1% penicillin/streptomycin and 10% FBS or 10% human serum. When reaching 70–80% confluence, adherent cells were gently washed with, and immediately cultured in, OPTI-MEM I before stimulating with crude LPS, purified SAA, in the absence or presence of 10Panx mimetic peptide. The cellular levels of Panx1, as well as the parallel extracellular release of HMGB1, NO, and other cytokines/chemokines were respectively determined by Western blotting analysis, Griess reaction, and Cytokine Antibody Arrays as previous described^8,53^. Because murine macrophages, human macrophages, and human PBMCs (monocytes) differed in their sensitivity to LPS or SAA stimulation, we used different ranges of optimal LPS and SAA concentrations to stimulate different types of innate immune cells.

### Hemichannel permeability assay

Lucifer yellow (LY) is often used to determine hemichannel permeability by the rate of tracer entry into the cells. As previously described^40^, the LY dye uptake assay relied on the capacity of this dye present in the extracellular space to cross the cytoplasmic membrane and became concentrated intracellularly, which could be quantified by fluorescence microscopy. To determine the effects of Panx1 mimetic peptides on hemichannel permeability, RAW264.7 cells were first subjected to a 16 h treatment with 1.0 µg/ml LPS before replacing the medium with fresh DMEM containing Panx1 mimetic peptides. Following a brief incubation (15 min), LY dye (1%) was added to the cell culture. Fifteen minutes later, cells were fixed with 2% paraformaldehyde, and the number of LY-positive cells was counted. The relative ratio of LY-positive cells to total cell counts (indicated by the DAPI staining) was used as the measurement of hemichannel permeability.

### Western Blotting

The cellular and extracellular levels of Panx1 in murine macrophage and human monocyte cultures were determined by Western blotting analysis using rabbit polyclonal or monoclonal antibodies against the N- or C-terminus. To normalize samples, an equal amount of cellular proteins or extracellular proteins in equal volume of culture medium conditioned by equal macrophage cell numbers was subjected to immunoblotting analyses. Proteins in equal volume of cell-conditioned culture medium was concentrated by ultrafiltration (with a MW cutoff of 10 kDa), and subsequently normalized to the same volume. Proteins in equal amount of total cellular protein or equal sample volume were resolved on sodium dodecyl sulfate (SDS)-polyacrylamide gels, and transferred to polyvinylidene difluoride (PVDF) membranes. After blocking with 5% nonfat milk, the membrane was incubated with respective antibodies (anti-Panx1, 1:1000; anti-β-actin. 1:5,000; anti-HMGB1, 1:1,000) overnight. Subsequently, the membrane was incubated with the appropriate secondary antibody, and the immune-reactive bands were visualized by chemiluminescence techniques. The relative band intensity was quantified using the UN-SCAN-IT Gel Analysis Software Version 7.1 (Silk Scientific Inc., Orem, UT, USA).

### Differential centrifugation

To characterize the properties of Panx1-containing structures in the macrophage-conditioned culture medium, cell supernatant was sequentially centrifuged at various speed (5000 × g, 10 min; 20,000 × g, 30 min), and proteins in each fractions were assayed for the presence of Panx1 by Western blotting analysis. Proteins in the initial cell-conditioned medium or the final supernatant of ultracentrifugation (20,000 × g) were concentrated by ultrafiltration using centricon-10 (with a molecular weight cutoff of 10 kDa) as previously described^54^.

### Panx1 immunostaining

Primary peritoneal macrophages were isolated from Balb/c mice as previously described^40^, stimulated with LPS or SAA for 6 h, and immunostained with rabbit polyclonal anti-Panx1 IgGs (1:1,000 dilution at 4 °C). Cells were fixed with 2% paraformaldehyde, permeabilized using 0.3% Triton X-100, and then incubated for 16 h with rabbit anti-Panx1 IgGs. After extensive washing, cells were incubated with donkey anti-rabbit Alexa 488 (Invitrogen) for 1 h at room temperature. The coverslips were then mounted on glass slides using anti-fade medium and examined under an epi-fluorescence microscope.

### ATP release assay

As previously described^40^, in addition to the LY dye uptake, we also used the ATP release assay to measure the Panx1 hemichannel activities. Briefly, RAW 264.7 cells were cultured in serum-free DMEM medium, and stimulated with crude LPS (1.0 μg/ml) in the absence or presence of Panx 1 mimetic peptide (100, 200 μg/ml). The cell-conditioned culture medium was collected and subjected to ATP measurement using the Molecular Probes® ATP Determination Kit (Cat. #A22066, Life Technologies). As previously described^40^, this method was based on luciferase’s absolute requirement for ATP in producing light (emission maximum ~560 nm at pH 7.8), to measure the extracellular ATP levels in LPS-stimulated RAW 264.7 cells.

### Cytokine Antibody Array

As previously described^40^, murine Cytokine Antibody Arrays (Cat. No. M0308003, RayBiotech Inc., Norcross, GA, USA), which simultaneously detect 62 cytokines on one membrane, were used to measure relative cytokine levels in macrophage-conditioned culture medium. Briefly, the membranes were incubated with equal volumes of culture medium, followed by sequential incubation with primary biotin-conjugated antibodies, and horseradish peroxidase–conjugated streptavidin. After exposing to X-ray film, the relative signal intensity was determined using the Scion Image software.

### Nitric Oxide (NO) Assay

The levels of nitric oxide in the culture medium were determined indirectly by measuring the NO^2−^ production with a colorimetric assay based on the Griess reaction as previously described^53^. NO^2−^ concentrations were determined with reference to a standard curve generated with sodium nitrite at various dilutions.

### Animal model of polymicrobial sepsis

This study was approved and performed in accordance with the guidelines of the Institutional Animal Care and Use Committee of the Feinstein Institute for Medical Research, Manhasset, New York, USA (Animal Protocol #2008-033, Approval date: November 7^th^, 2008). To evaluate the effect of Panx1 mimetic peptides on sepsis lethality, a clinically relevant animal model of sepsis induced by cecal ligation and puncture (CLP) was employed as previously described^40^. Briefly, the cecum of Balb/c mice was ligated at about 5 mm from the cecal tip, and then punctured once with a 22-gauge needle. 10Panx peptide and a scrambled control peptide were administered intraperitoneally into mice at indicated doses and time points, and animal survival rates were monitored for up to 2 wks.

### Real-time RT-PCR

Total RNA was isolated from various tissue using the Trizol reagent kit as per the manufacturer’s instructions, and reversely transcribed into the first-strand cDNA using the RevertAid™ First Strand cDNA Synthesis Kit. Following reverse transcription, a panel of established primers and probe for murine panx1 (Assay ID: Mm00450900_m1, Cat# 4351370, ThermoFisher Scientific), and glyceraldehyde 3-phosphate dehydrogenase gene (Assay ID: Mm99999915_g1, Cat.# 4331182, ThermoFisher Scientific) were used to quantify the Panx1 mRNA expression levels using a ABI 7900HT Fast Real-time PCR system (Applied Biosystems, Foster City, CA). Amplification was performed using the Taqman^®^ Universal Master Mix II with UNG Mastermix under the following conditions: 95 °C 10′; followed by 40 cycles of 95 °C for 15″ and 60 °C for 1′. The relative panx1 mRNA expression was calculated using the following formula:

$${\rm{\Delta }}{\rm{\Delta }}{\rm{C}}\,{\rm{e}}{\rm{x}}{\rm{p}}{\rm{r}}{\rm{e}}{\rm{s}}{\rm{s}}{\rm{i}}{\rm{o}}{\rm{n}}=2{\rm{\exp }}(\,-\,{\rm{\Delta }}{\rm{\Delta }}{\rm{C}}{\rm{t}}),$$

where ΔΔCt = ΔCt (treated group) − ΔCt (control group), ΔCt = Ct (target gene) − Ct (GAPDH), and Ct = cycle at which the threshold was reached. The relative abundance of Panx1 mRNA expression in control group was set as an arbitrary unit of 1, and the gene expression in treated groups was presented as folds of controls after normalization to GAPDH.

### Statistical analysis

One-way analyses of variance (ANOVA) followed by the Tukey test for multiple comparisons were used to compare between different groups. Student’s t-test was used for comparison between two groups. The Kaplan-Meier method was used to compare the differences in mortality rates between groups with log-rank post hoc test. A P value < 0.05 was considered statistically significant.

## Supplementary information

Enhanced Macrophage Pannexin 1 Expression and Hemichannel Activation Exacerbates Lethal Experimental Sepsis.
