# Supplementary material for: Enhanced Macrophage Pannexin 1 Expression and Hemichannel Activation Exacerbates Lethal Experimental Sepsis
Source: Sci Rep. 2019 Jan 17;9:160. doi: 10.1038/s41598-018-37232-z (PMC6336828; doi:10.1038/s41598-018-37232-z)
Supplement: Supplementary file 1 — Enhanced Macrophage Pannexin 1 Expression and Hemichannel Activation Exacerbates Lethal Experimental Sepsis. [file 41598_2018_37232_MOESM1_ESM.docx]

**Enhanced Macrophage Pannexin 1 Expression and Hemichannel Activation Exacerbates Lethal Experimental Sepsis.**

Weiqiang Chen ^1, #^, Shu Zhu ^1, #^, Yongjun Wang ^1^, Jianhua Li ^1^, Xiaoling Qiang ^1,2^, Xiaoling Zhao ^3^, Huan Yang ^1^, John D’Angelo ^1,2^, Lance Becker ^1,2^, Ping Wang ^1,2^, Kevin J. Tracey ^1,2^, and Haichao Wang ^1,2,*^

**^1^** The Feinstein Institute for Medical Research, Northwell Health, 350 Community Drive, Manhasset, NY 11030, USA; ^2^ Zucker School of Medicine at Hofstra/Northwell, 500 Hofstra Blvd, Hempstead, NY 11549, USA; ^3^ Department of Pathology, New York University School of Medicine, 550 1^st^ Ave, New York, NY 10016, USA

**Running title:** Pathogenic role of Pannexin 1 hemichannel in sepsis.

**Figure S1. LPS induced extracellular release of Panx1 by primary human monocytes.**  Peripheral blood mononuclear cells (HuPBMCs) were isolated from human blood by density gradient centrifugation through Ficoll, and stimulated with crude LPS at indicated concentrations for different time periods in the absence or presence of two anti-inflammatory agents, (-)-epigallocatechin gallate (**EGCG**) and tanshinone IIA sodium sulfonate (**TSN**). The extracellular Panx1 levels were determined by Western blotting analysis. Note that the first three lanes and the last lane was cropped out to make a composite **Figure 2C** to illustrate a time-dependent change of extracellular Panx1 levels and profiles.
